# Supplementary material for: Comparative Binding Ability of Human Monoclonal Antibodies against Omicron Variants of SARS-CoV-2: An In Silico Investigation
Source: Antibodies (Basel). 2023 Feb 23;12(1):17. doi: 10.3390/antib12010017 (PMC10045060; doi:10.3390/antib12010017)
Supplement: Supplementary file 1 [file antibodies-12-00017-s001.zip › antibodies-2151430-supplementary.pdf]

Supplementary materials

Table S1. Molecular docking studies of efficacious mAbs against the native spike protein of SARS-CoV-2.

Sl. No.	Native SARS-CoV-2	Interacting Monoclonal Antibody	Haddock 2.4 score
1.	Wild-type SARS-CoV-2	Bamlanivimab	**-88.6 +/- 5.5**
2.		Regdanvimab	**-129.5 +/- 10.6**
3.		Tixagevimab	-56.8 +/- 8.6
4.		Cilgavimab	**-89.3 +/- 9.6**
5.		Etesevimab	**-97.1 +/- 5.2**
6.		Casirivimab	-66.5 +/- 1.9
7.		Imdevimab	-80.2 +/- 6.4
8.		Sotrovimab	**-127.5 +/- 4.9**
9.		Adintrevimab	**-125.2 +/- 4.0**
10.		Beludavimab	**-102.6 +/- 7.5**
11.		Lomtegovimab	**-111.4 +/- 10.3**
12.		Romlusevimab	**-98.5 +/- 5.1**

Table S2. Molecular docking-based screening of the efficacious mAbs against the mutant spike protein from the Omicron variants of SARS-CoV-2 (Haddock score < -100.00).

Sl. No.	Lineage of SARS-CoV-2 Strain	Spike protein with mutation	Interacting Monoclonal Antibody	Haddock 2.4 score
**1.**	**B.1.1529 (Omicron)**	A67V	Bamlanivimab	-28.9 +/- 7.7
		D614G		-92.5 +/- 6.6
		D796K		-32.2 +/- 17.5
		E484A		-53.1 +/- 4.1
		G142D		-57.7 +/- 9.8
		G339D		-95.3 +/- 6.6
		G446S		-89.0 +/- 2.0
		G496S		-45.3 +/- 5.8
		H655Y		-8.1 +/- 23.4
		HV69-70del		22.5 +/- 14.6
		ins214EPE		-8.8 +/- 12.2
		**K417N**		**-106.7 +/-6.5**
		L212I		11.4 +/- 18.2
		L981F		-25.0 +/- 14.8
		N211del		-8.9 +/- 9.1
		N440K		-60.3 +/6.1
		N501Y		-91.8 +/- 6.8
		N679K		-2.7 +/- 35.6
		N764K		-20.4 +/- 5.8
		N856K		-22.9 +/- 4.3
		N969K		-40.4 +/- 25.4
		**P681H**		**-104.8 +/- 6.4**
		Q493R		-80.4 +/- 1.5
		Q498R		-41.0 +/- 6.1
		Q954H		-0.6 +/- 37.0
		**S371L**		**-106.4 +/- 6.2**
		S373P		-47.9 +/- 6.4
		S375F		-91.8 +/- 1.8
		S477N		-98.4 +/- 7.7
		T95I		-26.2 +/- 8.5
		T478K		-99.5 +/- 8.8
		T547K		-2.9 +/- 13.1
		VYY143-145		-22.6 +/- 24.4
		Y505H		-47.9 +/- 20.1
		MULTIVERSE		-69.2 +/- 4.4
2.		A67V	Regdanvimab	-81.2 +/- 6.1
		**D614G**		**-126.1 +/- 20.4**

	D796K		-92.5 +/- 11.0
	**E484A**		**-135.4 +/- 17.8**
	**G142D**		**-118.4+/-9.4**
	**G339D**		**-120.9 +/- 7.5**
	**G446S**		**-131.7 +/- 8.5**
	**G496S**		**-108.5 +/- 10.5**
	H655Y		-86.5 +/- 10.9
	HV69-70del		-37.7 +/- 9.0
	ins214EPE		-67.5 +/- 15.9
	**K417N**		**-122.4 +/- 7.9**
	L212I		-55.6 +/- 26.8
	L981F		-39.6 +/- 9.2
	N211del		-99.3 +/- 17.2
	**N440K**		**-118.0 +/- 17.7**
	**N501Y**		**-121.3±8.0**
	**N679K**		**-111.1 +/- 18.8**
	N764K		-54.8 +/- 7.8
	N856K		-17.3 +/-26.6
	N969K		-87.0 +/- 18.2
	**P681H**		**-137.2±8.1**
	**Q493R**		**-113.4 +/- 7.8**
	**Q498R**		**-131.3 +/- 9.6**
	Q954H		-57.1 +/- 12.5
	**S371L**		**-123.5 +/- 3.7**
	**S373P**		**-109.8 +/- 9.0**
	**S375F**		**-122.5 +/- 7.7**
	**S477N**		**-121.1 +/- 5.6**
	T95I		-3.3 +/- 55.7
	**T478K**		**-124.9 +/- 9.3**
	T547K		-49.2 +/- 8.0
	VYY143-145		-21.1 +/- 34.1
	**Y505H**		**-109.5 +/- 5.5**
	**MULTIVERSE**		**-147.6 +/- 13.5**
	A67V		-25.6 +/- 4.3
	D614G		-66.1+/-17.2
	D796K		-57.6 +/- 18.6
	E484A		-72.0 +/- 8.4
	**G142D**		**-112.0+/-14.4**
	G339D		-97.0 +/- 7.4
	G446S		-82.9 +/- 5.9
	G496S		-80.6 +/- 9.9
	H655Y		-41.9 +/- 39.8
	HV69-70del		-50.7 +/- 37.1
	ins214EPE		-77.2 +/- 18.0
	K417N		-87.3 +/-3.6
	L212I		-57.0 +/- 9.7
	L981F		-58.5 +/- 19.7
	N211del		17.0 +/- 23.3
3.	N440K	Cilgavimab	-60.5 +/- 5.0
	N501Y		-83.4+/-9.9
	N679K		-15.1 +/- 56.3
	N764K		-32.7 +/- 33.8
	N856K		-44.7 +/- 32.7
	N969K		-55.9 +/- 22.1
	P681H		-76.9+/-8.1
	Q493R		-84.5 +/- 5.7
	Q498R		-71.1 +/- 16.7
	Q954H		-84.5 +/- 5.7
	S371L		-73.1 +/- 16.7
	S373P		-64.7 +/- 12.4
	S375F		-72.8 +/- 4.3
	S477N		-68.0 +/- 12.1
	T95I		-87.4 +/- 5.8
	T478K		-83.3 +/- 5.9

	T547K		-66.9 +/- 26.8
	VYY143-145		-82.5 +/- 16.0
	Y505H		-67.5 +/- 15.9
	MULTIVERSE		-97.3 +/- 4.7
4.	A67V	Etesevimab	13.9 +/- 12.4
	**D614G**		**-139.0 +/- 3.2**
	D796K		24.7 +/- 13.5
	E484A		-23.9 +/- 11.9
	G142D		-48.8+/-2.6
	**G339D**		**-102.0 +/- 5.8**
	G446S		-95.2 +/- 2.7
	G496S		-27.0 +/- 7.5
	H655Y		24.0 +/- 14.5
	HV69-70del		-10.1 +/- 17.1
	ins214EPE		-0.5 +/- 11.8
	**K417N**		**-109.4 +/-3.7**
	L212I		-40.0 +/- 15.2
	L981F		14.9 +/- 6.3
	N211del		39.3 +/- 8.5
	N440K		-30.0 +/- 14.2
	N501Y		-58.1+/-13.8
	N679K		-30.2 +/- 7.1
	N764K		19.7 +/- 2.9
	N856K		12.0 +/- 15.6
	N969K		-18.1 +/- 6.3
	**P681H**		**-113.1+/-2.3**
	Q493R		-95.0 +/- 2.6
	Q498R		-41.0 +/- 4.6
	Q954H		35.2 +/- 17.1
	**S371L**		**-110.4 +/- 2.5**
	S373P		-37.1 +/- 4.1
	**S375F**		**-109.3 +/- 6.4**
	**S477N**		**-100.7 +/- 2.5**
	T95I		-28.3 +/- 22.6
	**T478K**		**-102.6 +/- 3.2**
	T547K		-25.9 +/- 43.3
	VYY143-145		-41.0 +/- 19.9
	Y505H		-39.5 +/- 9.3
	MULTIVERSE		-91.4 +/- 5.0
5.	A67V	Sotrovimab	-39.7 +/- 34.0
	D614G		-67.1+/-14.4
	D796K		7.5 +/- 46.3
	E484A		-95.2 +/- 9.5
	**G142D**		**-118.2+/-6.9**
	G339D		-78.8 +/- 6.4
	**G446S**		**-100.3 +/- 7.3**
	G496S		-86.9 +/- 6.7
	H655Y		0.4 +/- 53.2
	HV69-70del		-15.1 +/- 22.5
	ins214EPE		-6.2 +/- 46.3
	**K417N**		**-123.2 +/- 5.5**
	L212I		-26.5 +/- 28.1
	L981F		-71.8 +/- 11.3
	N211del		-46.5 +/- 4.4
	N440K		-26.5 +/- 13.1
	**N501Y**		**-112.3+/-2.7**
	N679K		-17.7 +/- 53.0
	N764K		-64.0 +/- 13.9
	N856K		-46.7 +/- 10.2
	N969K		-52.0 +/- 6.5
	**P681H**		**-129.0+/-14.7**
	**Q493R**		**-135.1 +/- 8.0**
	Q498R		-97.3 +/- 7.2
	Q954H		-45.0 +/- 47.7

	**S371L**		**-123.1 +/- 3.9**
	**S373P**		**-102.1 +/- 4.5**
	**S375F**		**-129.0 +/- 11.9**
	**S477N**		**-128.6 +/- 2.3**
	T95I		-30.9 +/- 49.5
	**T478K**		**-133.5 +/- 9.1**
	T547K		-56.9 +/- 20.2
	VYY143-145		-31.6 +/- 8.5
	**Y505H**		**-101.9 +/- 3.7**
	**MULTIVERSE**		**-108.9 +/- 10.1**
6.	A67V	Adintrevimab	-47.3 +/- 10.9
	**D614G**		**-119.9 +/- 2.6**
	D796K		-53.5 +/- 7.1
	E484A		-80.0 +/- 21.3
	**G142D**		**-112.2 +/- 11.4**
	**G339D**		**-113.7 +/- 5.7**
	**G446S**		**-110.9 +/- 5.0**
	G496S		-73.6 +/- 9.4
	H655Y		-51.2 +/- 24.3
	HV69-70del		-57.8 +/- 14.7
	ins214EPE		-56.2 +/- 6.3
	**K417N**		**-102.1 +/- 6.7**
	L212I		-49.7 +/- 16.0
	L981F		-53.5 +/- 4.1
	N211del		-30.7 +/- 20.1
	N440K		-59.4 +/- 19.0
	N501Y		-52.7 +/- 15.0
	N679K		-29.4 +/- 20.3
	N764K		-64.3 +/- 11.7
	N856K		-0.8 +/- 12.3
	N969K		-19.9 +/- 4.2
	**P681H**		**-118.2 +/- 8.0**
	**Q493R**		**-102.8 +/- 15.0**
	Q498R		-78.0 +/- 12.1
	Q954H		-12.4 +/- 7.3
	**S371L**		**-113.7 +/- 1.8**
	S373P		-96.8 +/- 5.6
	**S375F**		**-103.5 +/- 2.9**
	**S477N**		**-114.9 +/- 3.4**
	T95I		-35.2 +/- 13.7
	**T478K**		**-100.9 +/- 7.7**
	T547K		-45.0 +/- 6.9
	VYY143-145		-56.2 +/- 13.2
	Y505H		-69.0 +/- 19.0
	**MULTIVERSE**		**-110.0 +/- 3.6**
7.	A67V	Beludavimab	-75.4 +/- 13.4
	**D614G**		**-138.6 +/- 2.3**
	D796K		-40.5 +/- 14.1
	**E484A**		**-111.9 +/- 6.9**
	**G142D**		**-116.7 +/- 8.4**
	**G339D**		**-101.4 +/- 4.5**
	**G446S**		**-106.7 +/- 7.4**
	**G496S**		**-108.0 +/- 2.0**
	H655Y		-60.4 +/- 4.3
	HV69-70del		-23.1 +/- 15.7
	ins214EPE		-20.0 +/- 12.8
	K417N		-91.6 +/- 2.5
	L212I		-33.1 +/- 7.4
	L981F		-36.9 +/- 14.1
	N211del		7.2 +/- 34.8
	N440K		-86.5 +/- 5.7
	**N501Y**		**-105.0 +/- 6.4**
	N679K		26.5 +/- 10.9
	N764K		-15.4 +/- 47.1

	N856K		-37.1 +/- 7.6
	N969K		-40.1 +/- 13.7
	**P681H**		**-111.7 +/- 19.1**
	**Q493R**		**-106.1 +/- 4.6**
	Q498R		-98.2 +/- 3.4
	Q954H		2.9 +/- 25.1
	**S371L**		**-101.6 +/- 4.9**
	**S373P**		**-103.0 +/- 6.9**
	**S375F**		**-116.4 +/- 11.9**
	**S477N**		**-105.1 +/- 5.2**
	T95I		-42.3 +/- 12.5
	T478K		-99.1 +/- 5.5
	T547K		-7.7 +/- 39.4
	VYY143-145		-17.2 +/- 14.0
	**Y505H**		**-108.7 +/- 11.9**
	**MULTIVERSE**		**-129.8 +/- 1.8**
8.	A67V	Lomtegovimab	-35.8 +/- 6.6
	D614G		-88.1 +/- 4.0
	D796K		33.2 +/- 8.1
	E484A		-92.5 +/- 5.7
	G142D		-90.6 +/- 4.1
	G339D		-71.2 +/-10.2
	G446S		-75.3 +/- 25.5
	G496S		-68.8 +/- 4.7
	H655Y		21.3 +/- 36.4
	HV69-70del		54.4 +/- 6.2
	ins214EPE		60.2 +/- 10.6
	K417N		-85.4 +/- 21.3
	L212I		28.5 +/- 13.4
	L981F		31.1 +/- 13.0
	N211del		39.8 +/- 14.6
	N440K		-52.8 +/- 5.5
	N501Y		-48.4 +/- 20.7
	N679K		21.9 +/- 55.0
	N764K		38.8 +/- 7.4
	N856K		23.0 +/- 5.4
	N969K		35.5 +/- 6.7
	P681H		-95.7 +/- 17.2
	**Q493R**		**-123.9 +/- 5.2**
	Q498R		-62.3 +/- 4.5
	Q954H		26.6 +/- 4.3
	S371L		-81.5 +/- 12.5
	S373P		-76.0 +/- 19.9
	**S375F**		**-103.1 +/- 8.7**
	**S477N**		**-125.1 +/- 3.6**
	T95I		27.1 +/- 7.2
	**T478K**		**-103.4 +/- 3.6**
	T547K		31.1 +/- 45.9
	VYY143-145		34.2 +/- 32.1
	Y505H		-67.9 +/- 5.9
	**MULTIVERSE**		**-112.4 +/- 2.4**
9.	A67V	Romlusevimab	3.2 +/- 8.3
	**D614G**		**-104.5 +/- 3.7**
	D796K		3.3 +/- 11.5
	E484A		-47.1 +/- 9.8
	G142D		-36.3 +/- 11.7
	**G339D**		**-108.6 +/- 1.8**
	G446S		-85.2 +/- 8.5
	G496S		-51.2 +/- 5.5
	H655Y		14.5 +/- 7.4
	HV69-70del		-23.3 +/- 58.6
	ins214EPE		14.5 +/- 2.0
	K417N		-44.8 +/- 6.5
	L212I		-18.1 +/- 5.0

L981F	18.1 +/- 3.6
N211del	9.4 +/- 3.8
N440K	-47.0 +/- 3.5
N501Y	-44.7 +/- 5.9
N679K	-15.3 +/- 28.6
N764K	22.5 +/- 7.6
N856K	6.5 +/- 6.5
N969K	7.6 +/- 8.8
P681H	-74.2 +/- 2.7
Q493R	-95.2 +/- 8.7
Q498R	-45.0 +/- 4.3
Q954H	22.4 +/- 4.6
S371L	-86.3 +/- 4.9
S373P	-35.3 +/- 8.0
**S375F**	**-103.0 +/- 6.5**
S477N	-95.5 +/- 8.3
T95I	5.1 +/- 17.1
T478K	-74.5 +/- 15.3
T547K	31.3 +/- 7.8
VYY143-145	11.9 +/- 23.4
Y505H	-60.5 +/- 20.5
MULTIVERSE	-95.3 +/- 6.7

Table S3. List of all the effective mAbs and their level of efficacy against B.1.1529 (Omicron) variants.

Sl. No.	Lineage of SARS-CoV-2 Strain	Spike protein with mutation	Interacting Monoclonal Antibody	Binding Affinity ΔG (kcal mol^{-1})	Haddock 2.4 score
	B.1.1529 (Omicron)	K417N	Bamlanivimab	-11.1	-106.7±6.5
		P681H		-11.9	-104.8±6.4
		S371L		-11.1	-106.4±6.2
		**D614G**	**Regdanivimab**	-13.9	-126.1±20.4
		**E484A**		-15.7	-135.4±17.8
		G142D		-9.3	-118.4±9.4
		**G339D**		-10.0	-120.9±7.5
		**G446S**		-12.3	-131.7±8.5
		G496S		-9.7	-108.5±10.5
		**K417N**		-11.1	-122.4±7.9
		**N440K**		**-15.8**	-118.0±17.7
		**N501Y**		-13.7	-121.3±8.0
		**N679K**		-14.9	-111.1±18.8
		**P681H**		-10.3	-137.2±8.1
		**Q493R**		-10.5	-113.4±7.8
		**Q498R**		-14.6	-131.3±9.6
		**S371L**		-10.9	-123.5±3.7
		**S373P**		-8.9	-109.8±9.0
		**S375F**		-10.2	-122.5±7.7
		**S477N**		-11.0	-121.1±5.6
		**T478K**		-12.8	-124.9±9.3
		**Y505H**		-13.9	-109.5±5.5
		**MULTIVERSE**		-13.0	-147.6±13.5
		G142D	Cilgavimab	**-18.6**	-112.0±14.4
		D614G	Etesevimab	-10.9	-139.0±3.2
		G339D		-12.8	-102.0±5.8
		K417N		-14.1	-109.4±3.7
		P681H		-12.6	-113.1±2.3
		S371L		-11.4	-110.4±2.5
		S375F		-14.3	-109.3±6.4
		S477N		-13.3	-100.7±2.5
		T478K		-12.7	-102.6±3.2

	G142D	Sotrovimab	-12.0	-118.2±6.9
	G446S		-10.6	-100.3±7.3
	K417N		-11.9	-123.2±5.5
	N501Y		-13.6	-112.3±2.7
	P681H		-12.7	-129.0±14.7
	Q493R		-12.2	-135.1±8.0
	S371L		-10.9	-123.1±3.9
	S373P		-10.2	-102.1±4.5
	S375F		-11.7	-129.0±11.9
	S477N		-8.6	-128.6±2.3
	T478K		-11.4	-133.5±9.1
	Y505H		-10.6	-101.9±3.7
	MULTIVERSE		-9.7	-108.9±10.1
	**D614G**	**Adintrevimab**	-13.5	-119.9±2.6
	**G142D**		-13.6	-112.2±11.4
	**G339D**		**-15.5**	-113.7±5.7
	**G446S**		-14.7	-110.9±5.0
	**K417N**		-14.2	-102.1±6.7
	**P681H**		-14.3	-118.2±8.0
	**Q493R**		-10.8	-102.8±15.0
	**S371L**		-14.3	-113.7±1.8
	**S375F**		-15.1	-103.5±2.9
	**S477N**		-14.7	-114.9±3.4
	**T478K**		-14.6	-100.9±7.7
	**MULTIVERSE**		-12.2	-110.0±3.6
	**D614G**	**Beludavimab**	-13.9	-138.6±2.3
	**E484A**		-12.7	-111.9±6.9
	**G142D**		-11.5	-116.7±8.4
	**G339D**		-10.7	-101.4±4.5
	**G446S**		-13.6	-106.7±7.4
	**G496S**		-10.7	-108.0±2.0
	**N501Y**		-12.7	-105.0±6.4
	**P681H**		-9.4	-111.7±19.1
	**Q493R**		-13.6	-106.1±4.6
	**S371L**		**-15.0**	-101.6±4.9
	**S373P**		-12.6	-103.0±6.9
	**S375F**		-12.8	-116.4±11.9
	**S477N**		-13.8	-105.1±11.9
	**Y505H**		-13.0	-108.7±11.9
	**MULTIVERSE**		-12.3	-129.8±1.8
	Q493R	Lomtegovimab	-14.8	-123.9±5.2
	S375F		-13.7	-103.1±8.7
	S477N		-14.7	-125.1±3.6
	T478K		-14.1	-103.4±3.6
	MULTIVERSE		-15.6	-112.4±2.4
	D614G	Romlusevimab	**-15.3**	-104.5±3.7
	G339D		-12.7	-108.6±1.8
	S375F		-14.7	-103.0±6.5

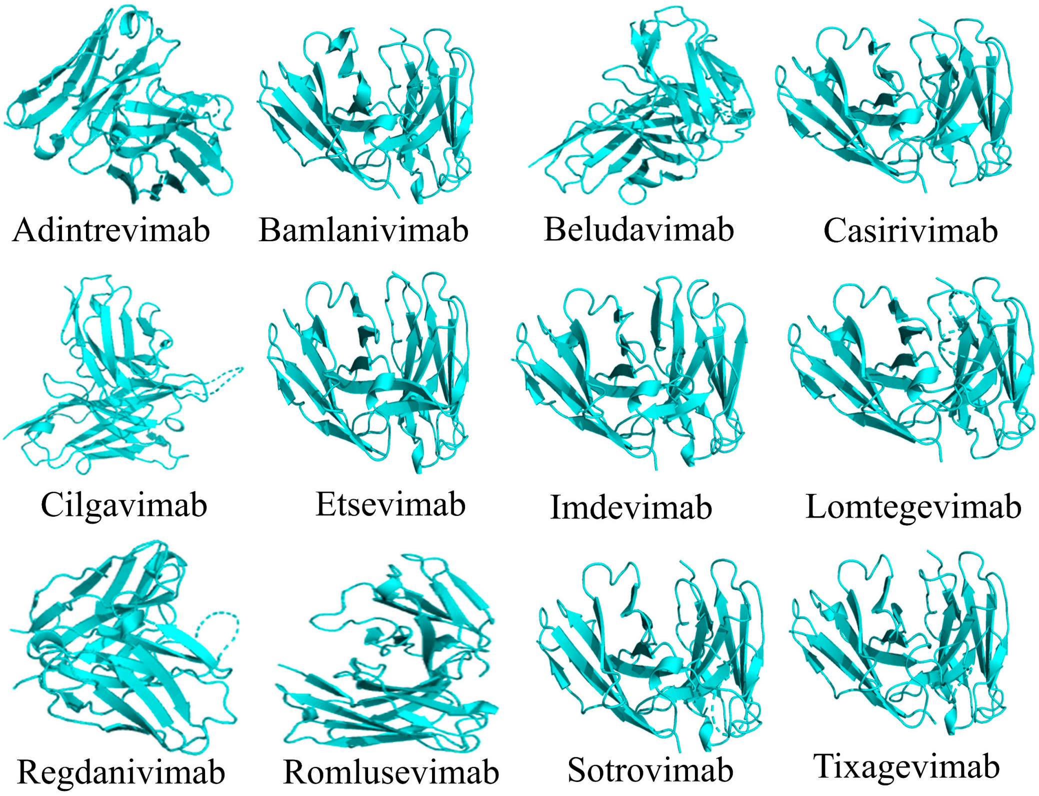

Figure S1. 3D conformation of all the 12 mAbs that were utilized in this study.

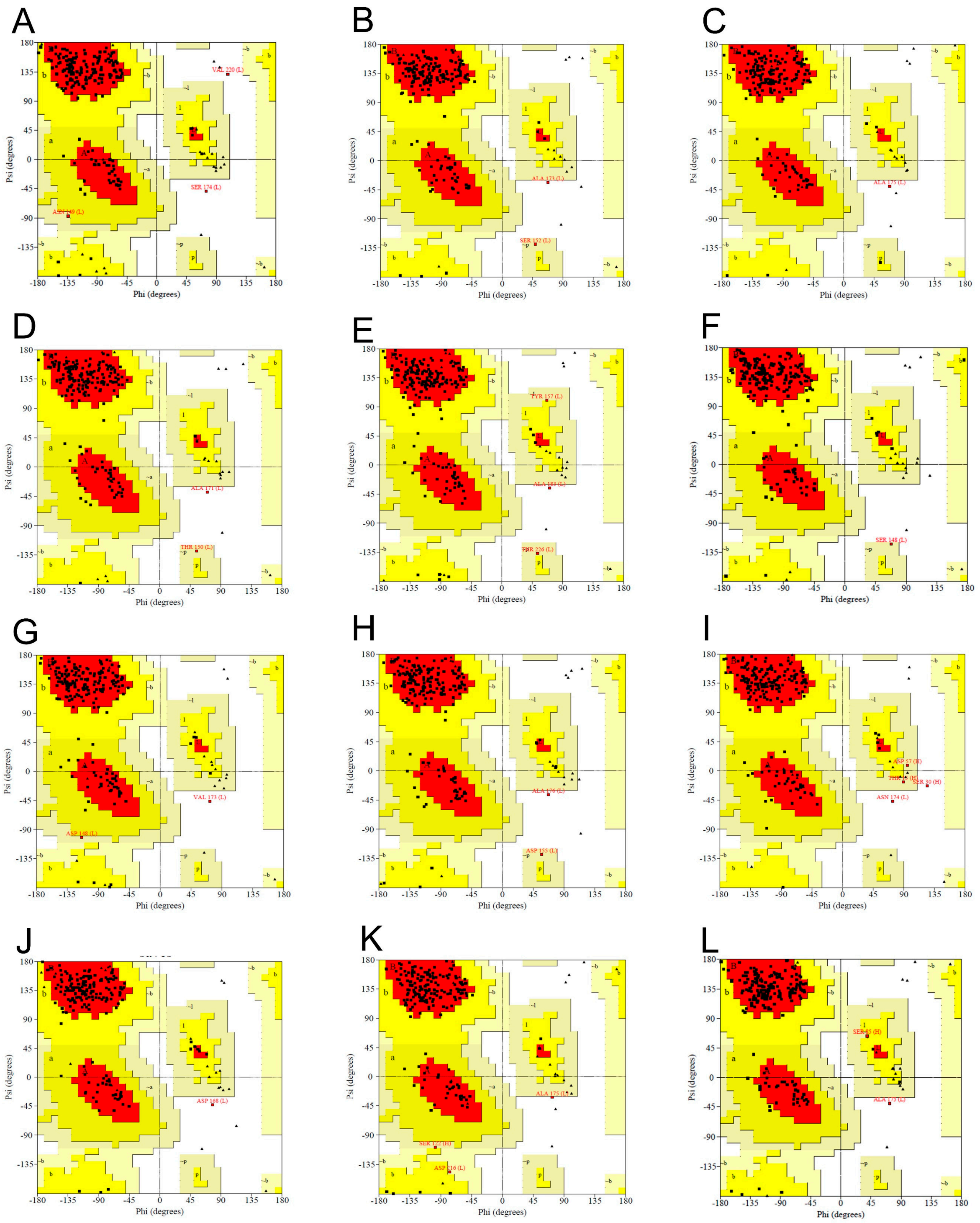

Figure S2. Ramachandran plots of 12 mAbs that indicate their different stereochemical stability. A. Adintrevimab, B. Bamlanivimab, C. Beludavimab, D. Casirivimab, E. cilgavimab, F. Etesevimab, G. Imdevimab, H. Lomtegovimab, I. Regdanvimab, J. romlusevimab, K. sotrovimab, L. Tixagevimab.

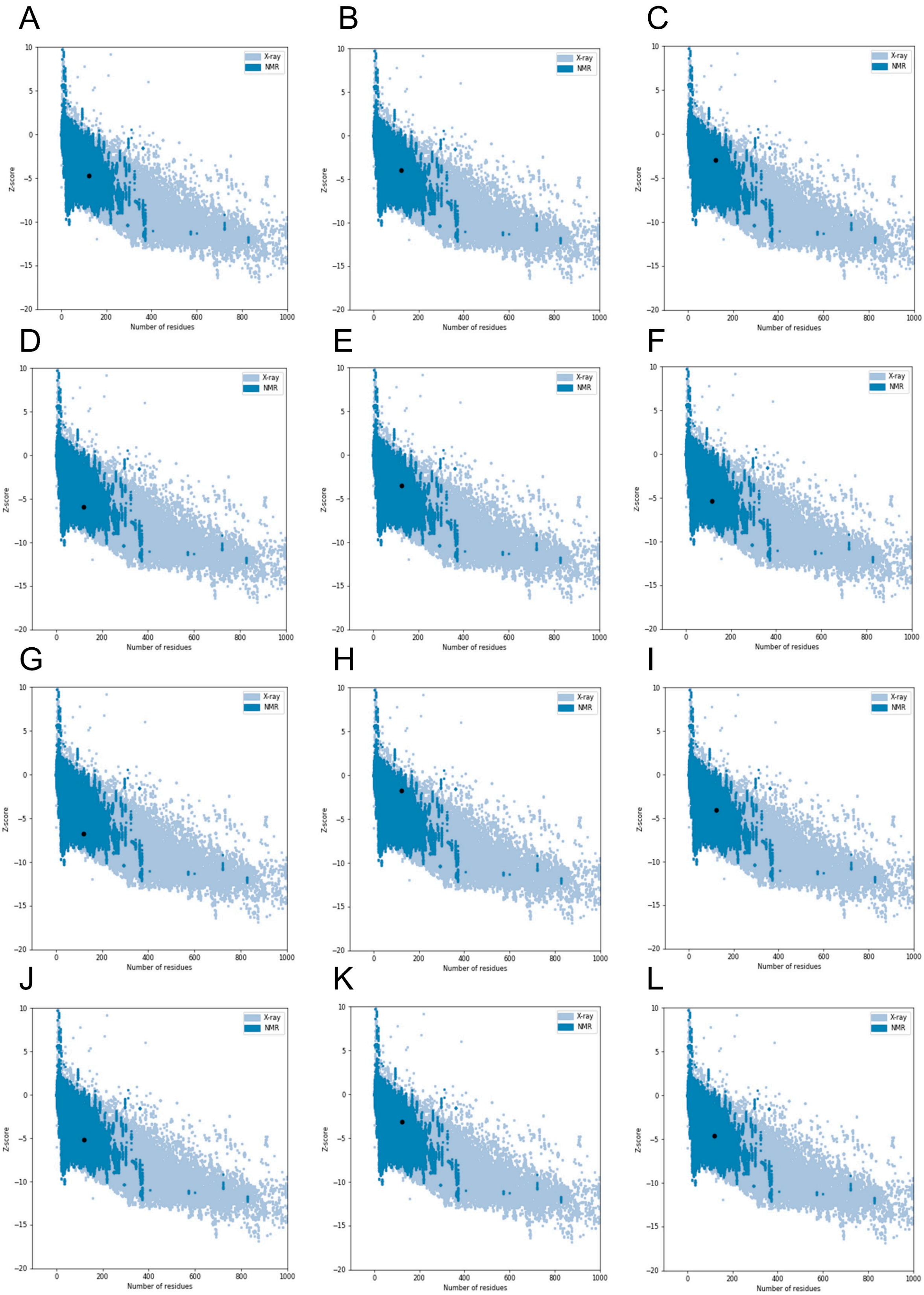

Figure S3. Z-plot with significant Z scores that reflect good stereochemical quality of the 12 mAbs. A. Adintrevimab, B. Bamlanivimab, C. Beludavimab, D. Casirivimab, E. cilgavimab, F. Etesevimab, G. Imdevimab, H. Lomtegovimab, I. Regdanvimab, J. romlusevimab, K. sotrovimab, L. Tixagevimab.

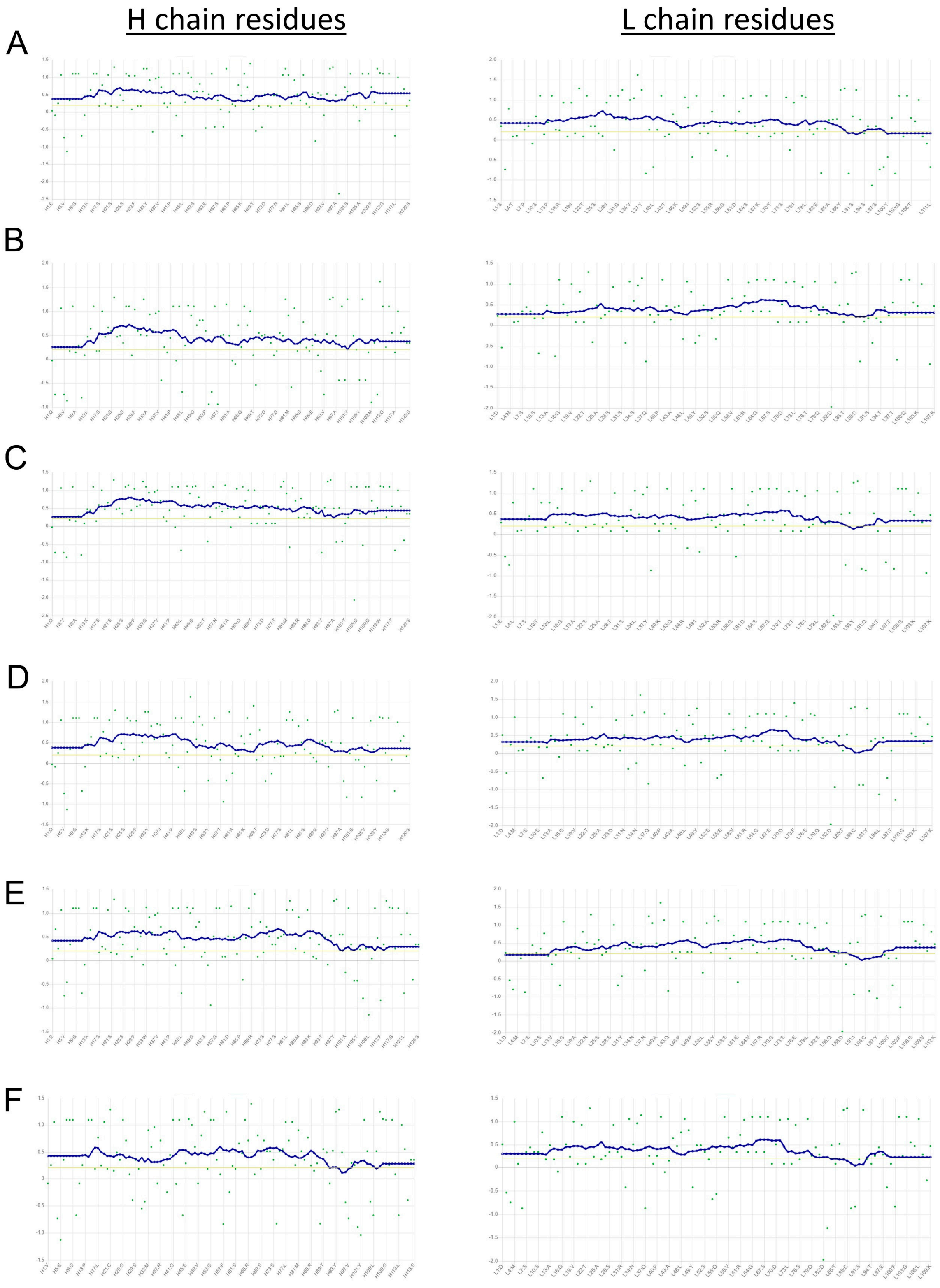

Figure S4. Verify 3D plots of 12 mAbs that describe the nature of 3D conformation. A. Adintrevimab, B. Bamlanivimab, C. Beludavimab, D. Casirivimab, E. cilgavimab, F. Etesevimab, G. Imdevimab, H. Lomtegovimab, I. Regdanvimab, J. romlusevimab, K. sotrovimab, L. Tixagevimab.

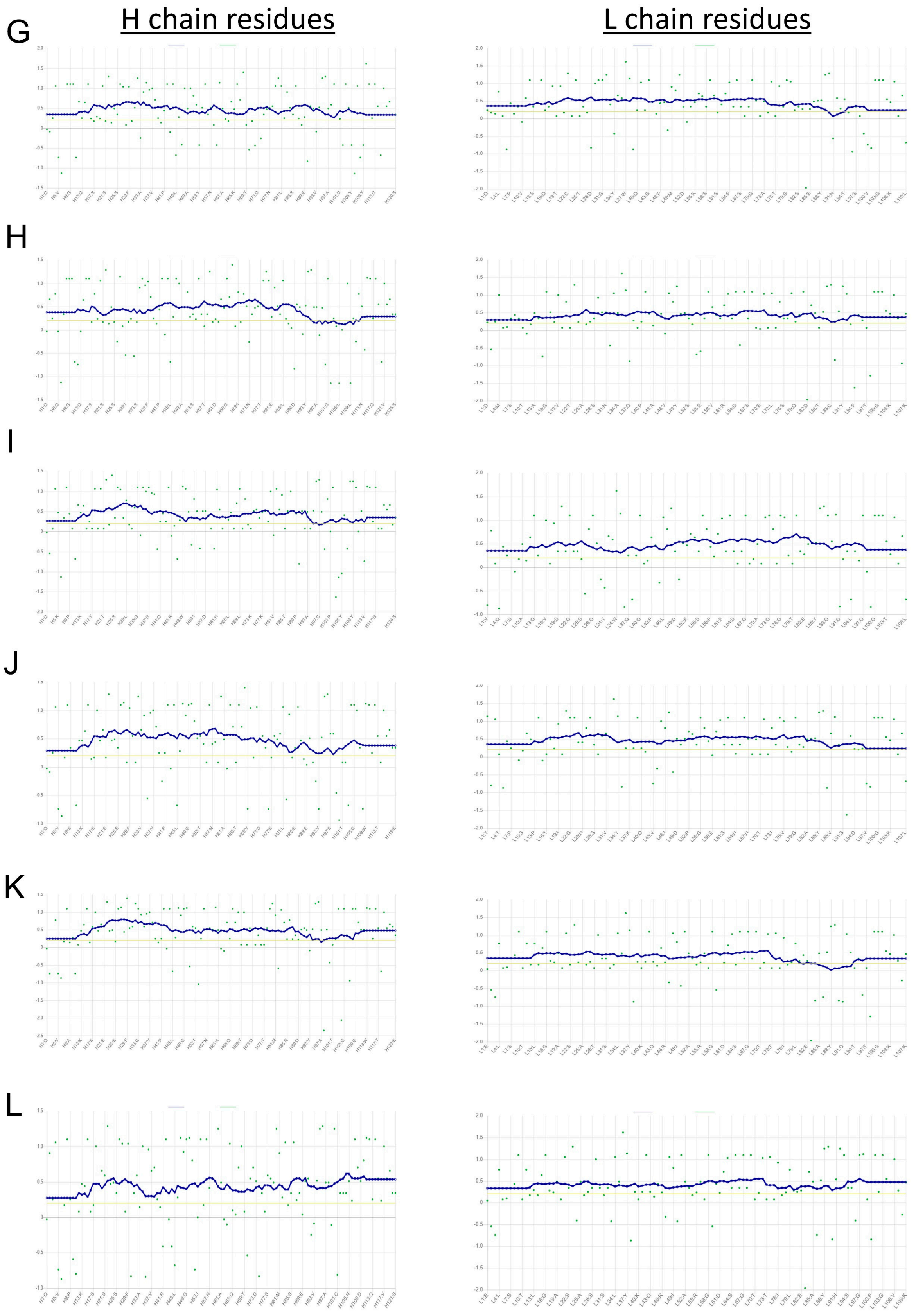

Figure S4. (contd.). Verify 3D plots of 12 mAbs that describe the nature of 3D conformation of them respectively. A. Adintrevimab, B. Bamlanivimab, C. Beludavimab, D. Casirivimab, E. cilgavimab, F. Etesevimab, G. Imdevimab, H. Lomtegovimab, I. Regdanvimab, J. romlusevimab, K. sotrovimab, L. Tixagevimab.

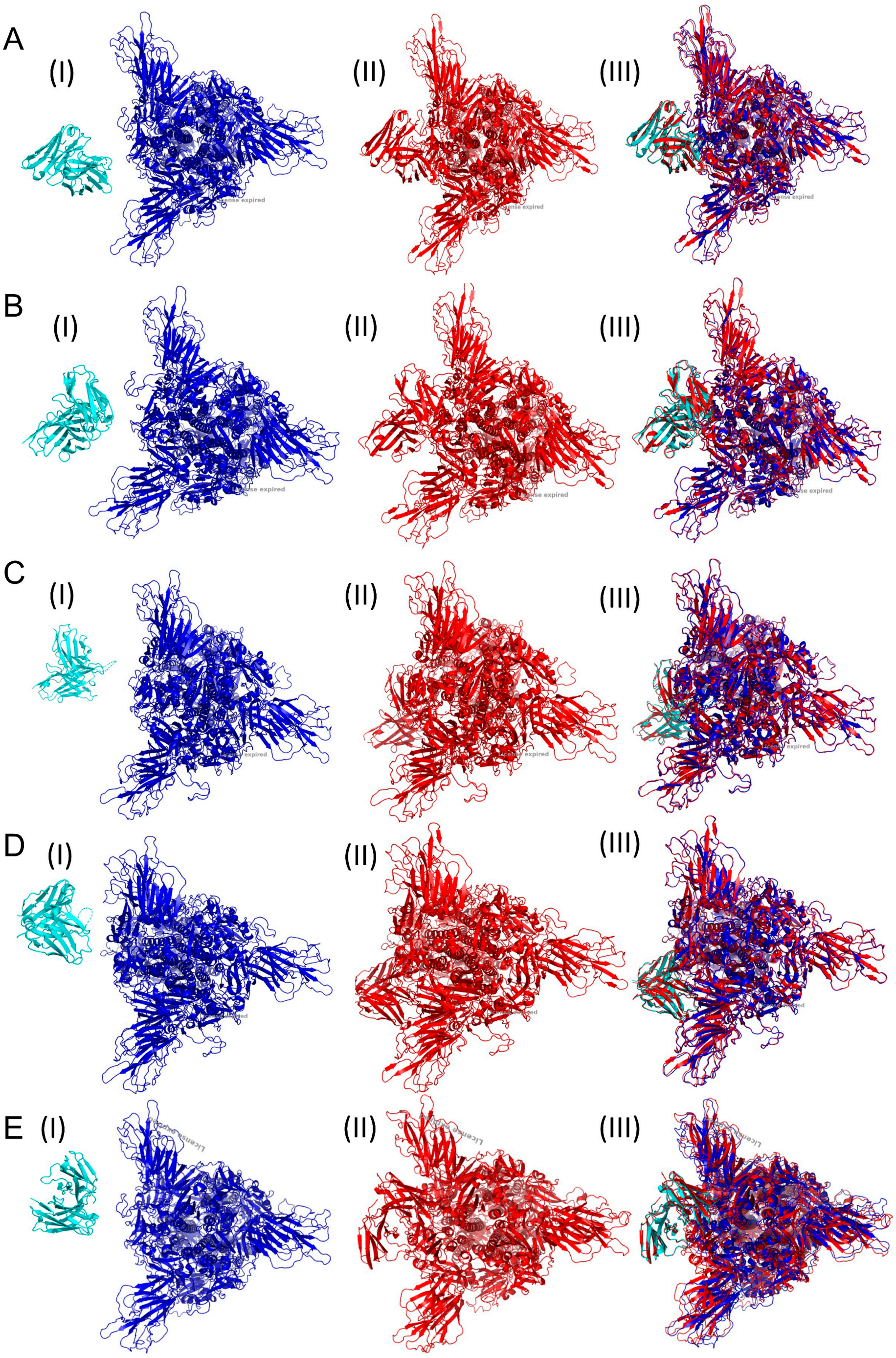

Figure S5. Conformational changes of the mAbs and the spike proteins of the Omicron due to the formation of mAb-spike protein complex. A. 3D structure of (i) unbound adintrevimab and G339D mutant spike protein of Omicron (shown in cyan and blue, respectively) and (ii) adintrevimab_G339D complex (shown in red). (iii) Superimposed bound and unbound forms of adintrevimab and G339D mutant spike protein. B. 3D structure of (i) unbound beludavimab and S371L mutant spike protein of Omicron (shown in cyan and blue, respectively) and (ii) beludavimab_S371L complex (shown in red). (iii) Superimposed bound and unbound forms of beludavimab and S371L mutant spike protein. C. 3D structure of (i) unbound cilgavimab and G142D mutant spike protein of Omicron (shown in cyan and blue, respectively) and (ii) cilgavimab_G142D complex (shown in red). (iii) Superimposed bound and unbound forms of cilgavimab_G142D mutant spike protein. D. 3D structure of (i) unbound regdanivimab and N440K mutant spike protein of Omicron (shown in cyan and blue, respectively) and (ii) regdanivimab_N440K complex (shown in red). (iii) Superimposed bound and unbound forms of regdanivimab and N440K mutant spike protein. E. 3D structure of (i) unbound romlusevimab and D614G mutant spike protein of Omicron (shown in cyan and blue, respectively) and (ii) romlusevimab_D614G complex (shown in red). (iii) Superimposed bound and unbound forms of romlusevimab and D614G mutant spike protein.

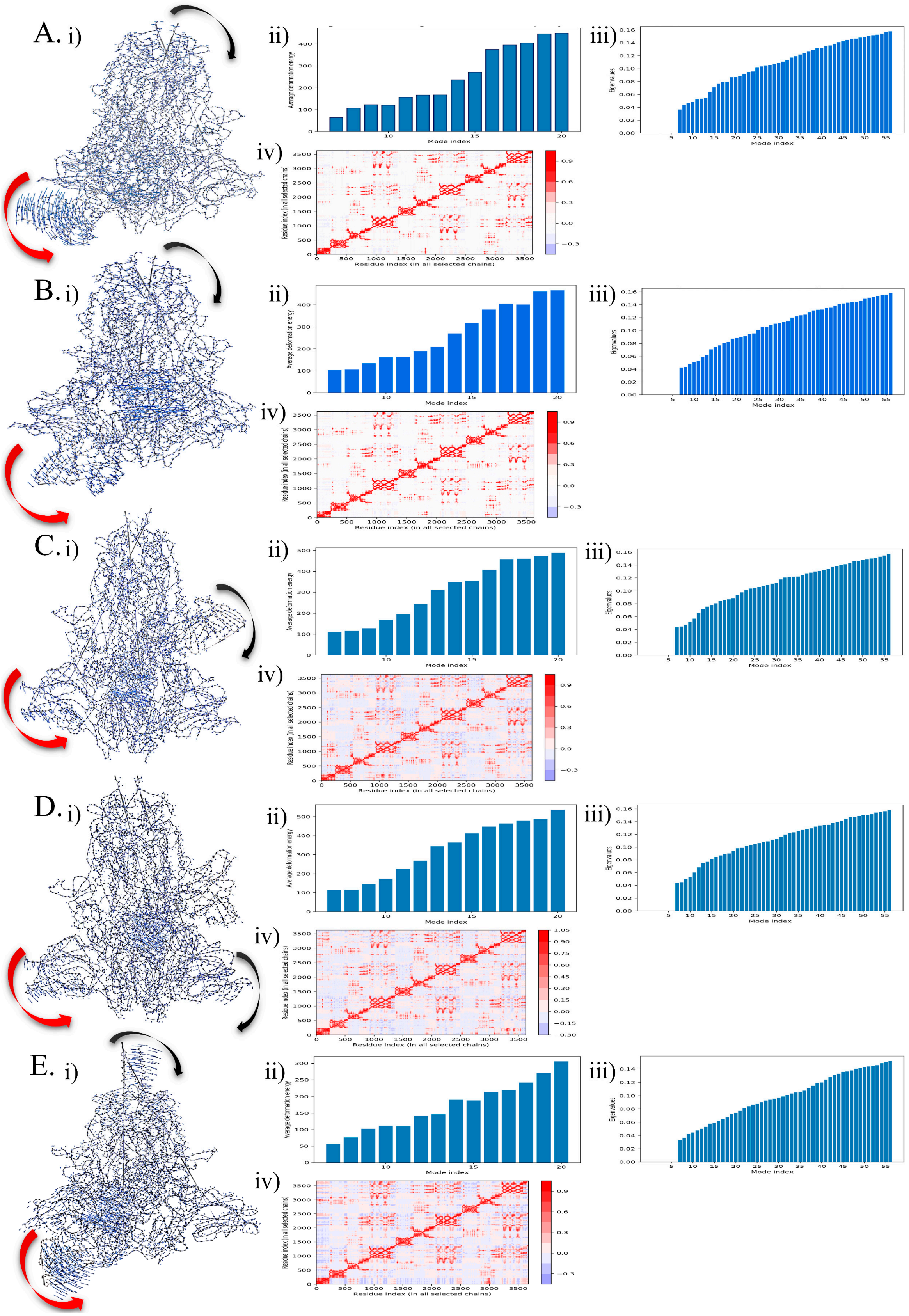

Figure S6. Comparative analyses of normal modes of 5 mAb-omicron spike protein complexes. A. adintrevimab_G339D, B. beludavimab_S371L, C. cilgavimab_G142D, D. regdanivimab_N440K and E. romlusevimab_D614G respectively. (i). Visualization of molecular motion. (ii). Illustration of deformation energies of different modes. (iii). Eigenvalues. (iv). Correlation matrix that facilitates understanding of correlated (shown in red), uncorrelated (shown in white) and anti-correlated (shown in blue) motions of Cα atoms.

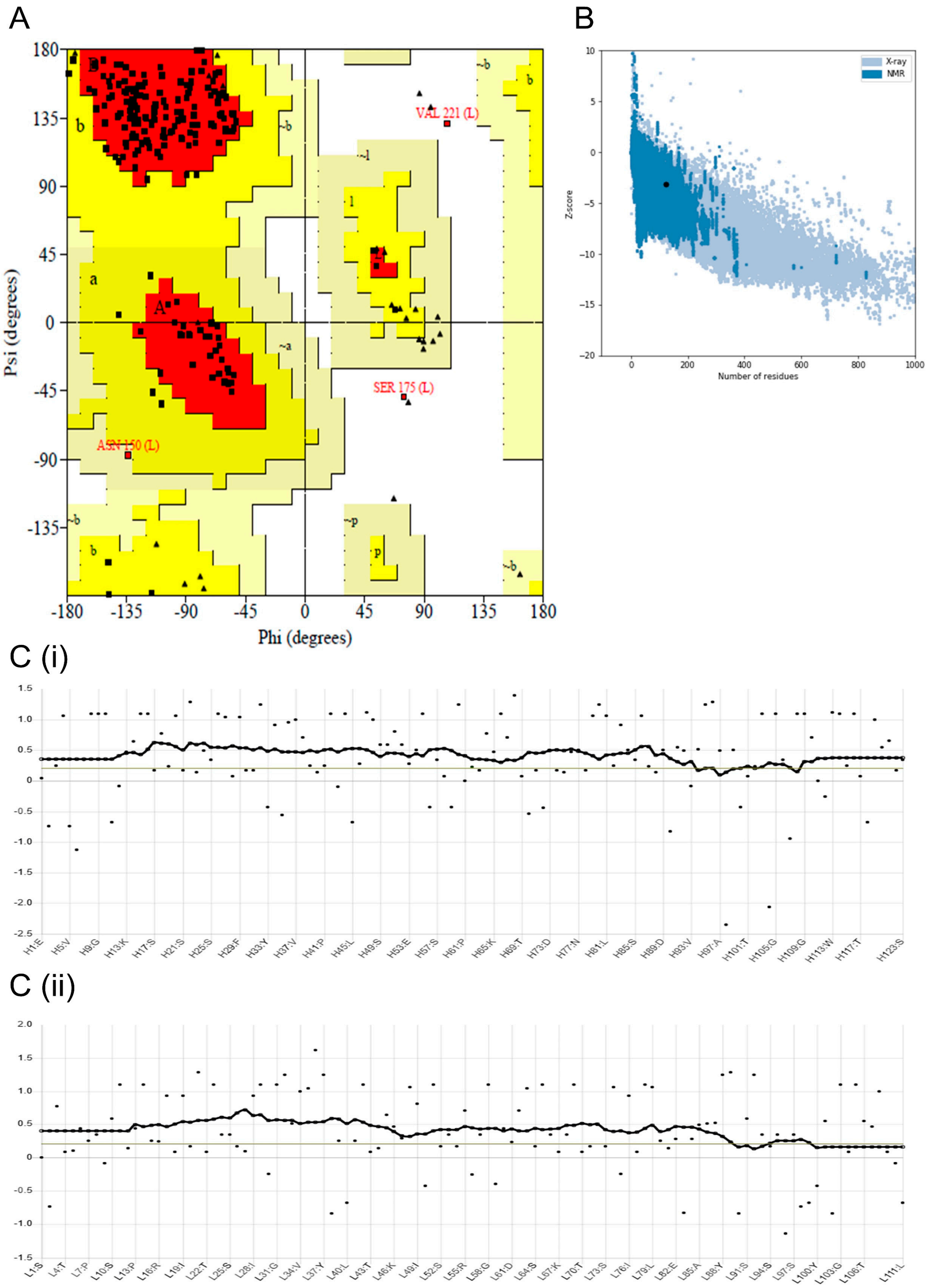

Figure S7. Stereochemical quality of the 3D conformation of the Adintrevimab-Framework-Beludavimab-CDRH3 chimeric mAb by A. Ramachandran plot, B. Z-Score, and, C. VERIFY 3D (where C(i) is directing heavy chain and C(ii) light chain).

Amino acid fasta sequence of the adintrevimab-framework-beludavimab-CDRH3 chimeric antibody

>ADIN_FM_BELU_CDRH3_Chimeric_H
EVQLVESGGGLVKPGGSLRLSCAASGFTFSSYYMNWVRQAPGKGLE
WVSSISEDGYSTYYPDSLKGRFTISRDSAKNSLYLQMNSLRADDTAV
YYCARDYTRGWGSIGGFDNWGQGTLVTVSS
>ADIN_FM_BELU_CDRH3_Chimeric_L
SVLTQPPSVSGAPGQRITISCTGSSSNIGAGYDVHWYQQLPGTAPKLL
IYGSSSRNSGVPDRFSGSKSGTSASLAITGLQAEDEADYYCQSYDSSL
SVLYTFGTGTKVTVL

Deduced nucleic acid sequence of the adintrevimab-framework-beludavimab-CDRH3 chimeric antibody

> Adintrevimab-framework-Beludavimab-CDRH3
gargtncarytngtngarwsnggnggnggnytngtnaarccnggnggnwsnytnmgnytnwsntg
ygcngcnwsnggnttyacnttywsnwsntaytayatgaaytgggtnmgncargcnccnggnaargg
nytngartgggtnwsnwsnathwsngargayggntaywsnacntaytayccngaywsnytnaargg
nmgnttyacnathwsnmgngaywsngcnaaraaywsnytntayytncaratgaaywsnytnmgn
gcngaygayacngcngtntaytaytgygcnmgngaytayacnmgnggntggggnwsnathggng
gnttygayaaytggggncarggnacnytngtnacngtnwsnwsnwsngtnytnacncarccnccnw
sngtnwsnggngcnccnggncarmgnathacnathwsntgyacnggnwsnwsnwsnaayathg
gngcnggntaygaygtncaytggtaycarcarytnccnggnacngcnccnaarytnytnathtayggn
wsnwsnwsnmgnaaywsnggngtnccngaymgnttywsnggnwsnaarwsnggnacnwsng
cnwsnytngcnathacnggnytncargcngargaygargcngaytaytaytgycarwsntaygayws
nwsnytnwsngtnytntayacnttyggnacnggnacnaargtnacngtnytn

Figure S8. Amino acid and deduced nucleic acid sequences of Adintrevimab-Framework-Beludavimab-CDRH3 chimeric mAb.

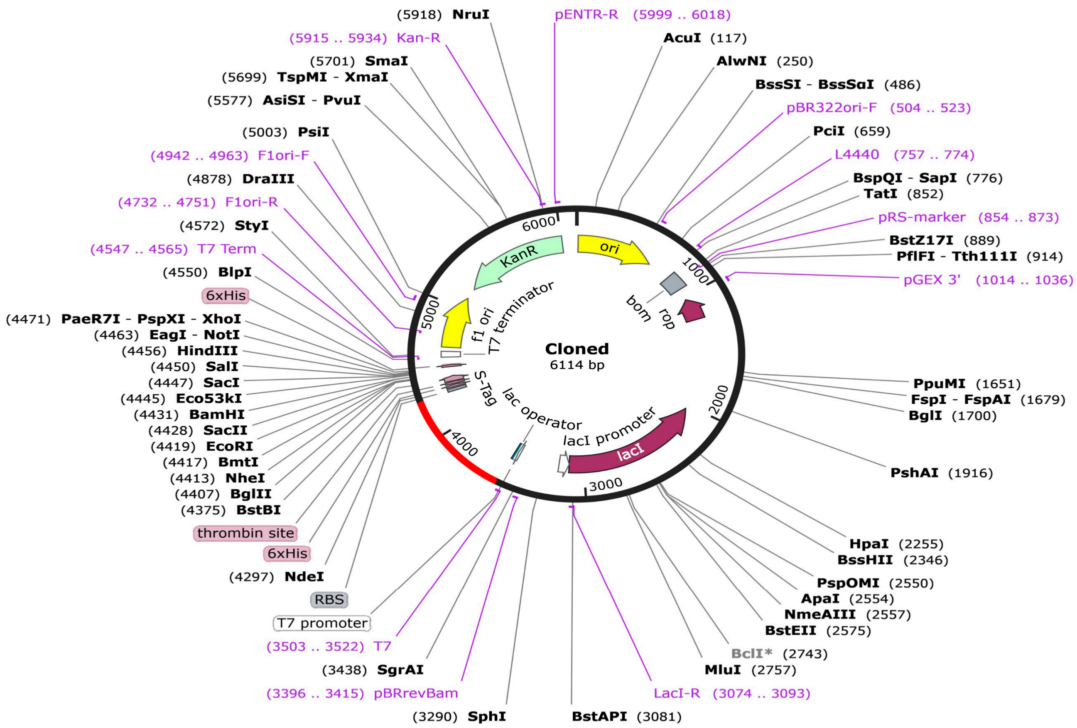

Figure S9. In-silico cloning of a chimeric mAb. Gene sequence of Adintrevimab-Framework-Beludavimab- CDRH3 chimeric mAb was cloned into pET30ax expression vector. The mAb region is represented by red color. We performed reverse translation and codon optimization of the chimeric mAb employing Java Codon Adaptation Tool (JCat) (http://www.jcat.de/Start.jsp, accessed on 6 June 2022) for the therapeutic and safety validation. Next, we explored Addgene database (https://www.addgene.org/, accessed on 6 June 2022) for screening the most suitable prokaryotic expression vector and the cDNA of the chimeric mAb was eventually cloned in-silico using the molecular cloning tool SnapGene tool (https://www.snapgene.com/, accessed on 6 June 2022). Recombinant production of mAb is crucial for the validation of the in-silico findings. Keeping this in mind, we transformed the amino acid sequence of heavy and light chain of Adintrevimab-Framework-Beludavimab-CDRH3 chimeric mAb in FASTA format and the cDNA sequence was deduced following codon optimization to express in E. coli K12 strain. XbaI restriction sites were added at both 5′ and 3′ terminal of mAb cDNA that facilitated the cloning in pET30ax vector (5406 bp) and construction of the recombinant plasmid. The modified cloned gene for expressing the recombinant mAb is 708bp long.
